# Characterization of membrane protein–nanobody complexation *via* flow-induced dispersion analysis (FIDA)

**DOI:** 10.1039/d6an01036a

**Published:** 2026-10-29

**Authors:** Kiefer O. Ramberg, Monika Olejnik, Saúl Gallego-Martínez, Andrew Quigley, Martin Caffrey

**Affiliations:** a School of Chemical Sciences, Dublin City University Glasnevin Dublin 9 D09 V209 Ireland kiefer.ramberg@dcu.ie; b School of Biochemistry & Immunology, Trinity College Dublin Dublin D02 R590 Ireland matin.caffrey@tcd.ie; c Astbury Centre for Structural Molecular Biology, School of Molecular and Cellular Biology, Faculty of Biological Sciences, University of Leeds UK; d Membrane Protein Laboratory, Diamond Light Source, Research Complex at Harwell Didcot UK

## Abstract

Nanobody binders may be raised against membrane protein targets for a myriad of applications including the modulation of bioactivity and to aid in structural elucidation. Flow-Induced Dispersion Analysis (FIDA) is a burgeoning analytical technique which has been used to quantify protein–protein binding interactions. FIDA offers a straightforward route to characterizing membrane protein–nanobody complexation in terms of affinity and particle size. A protocol for the rapid characterisation of nanobodies that bind membrane proteins with high affinity using FIDA would expedite the development of novel nanobody-based biomedicines and facilitate nanobody-assisted membrane protein structural biology. In this study, FIDA was used to characterize the complexation of a nanobody and a model membrane integral protein transporter. To this end, the nanobody was fluorescently labelled using fluorescein isothiocyanate. A simple two-step protocol for producing labelled nanobody stocks devoid of interfering free dye is presented. FIDA experiments entailing sequential addition of the micellarised transporter protein to the labelled nanobody in detergent-containing buffer revealed single digit nanomolar binding consistent with binding constants obtained previously by surface plasmon resonance analysis.

## Abstract 2

Nanobody binders are routinely raised against membrane protein targets. Here, flow-induced dispersion analysis (FIDA) is used to characterize the binding of a nanobody to a membrane protein transporter complex with nanomolar affinity.

## Graphical abstract

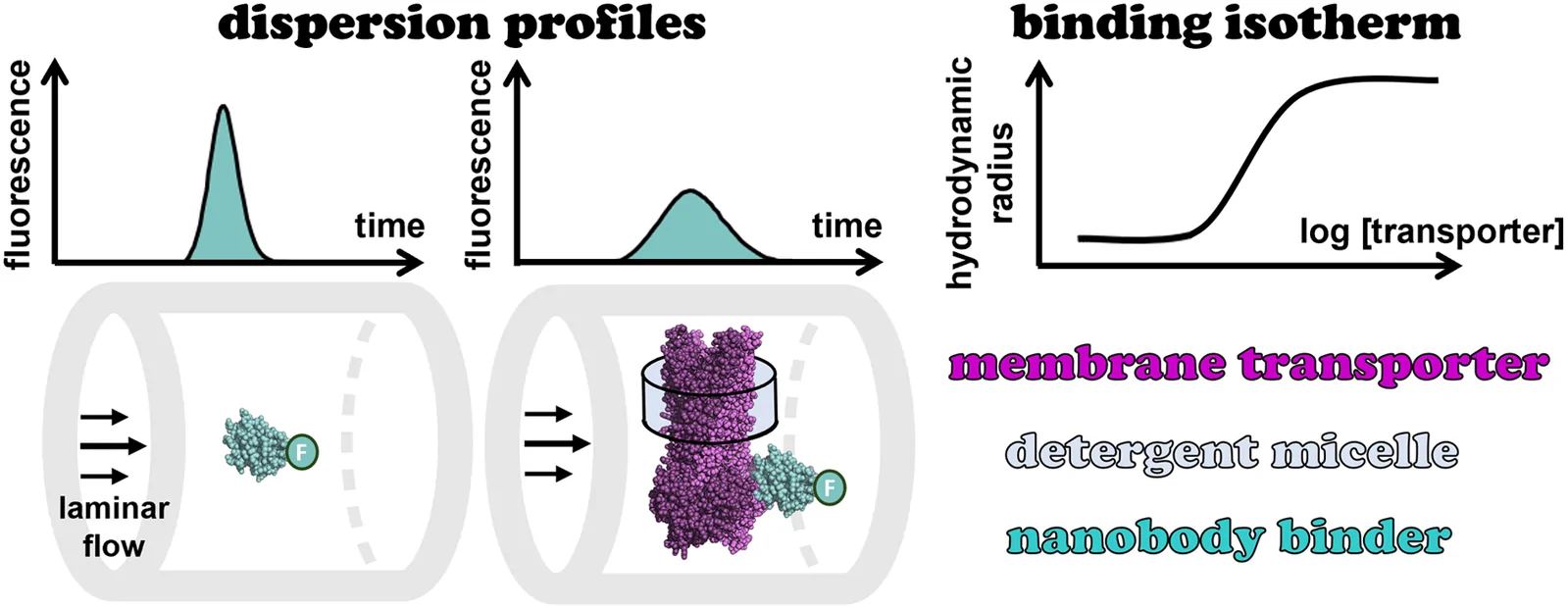

Membrane proteins are the most intensively studied targets in drug discovery.^1–3^ High-resolution membrane protein structures obtained by macromolecular X-ray crystallography (MX) and cryo-electron microscopy (cryo-EM) underpin most drug discovery efforts.^1,3^ MX requires stable protein and structure-quality crystals that can be challenging to grow. Stable proteins are also required for cryo-EM which, in addition, has a requirement for targets with a molecular weight in excess of ∼40 kDa.^1,4^ These issues may be remedied by using highly specific protein binders known as nanobodies.^5–9^ Nanobodies are small, stable single-chain antibodies derived from the minimalist immunomodulatory molecules of camelids. Nanobodies can be generated against membrane protein targets by camelid immunization^5^ and by display methods^6,7^ with coverage across a wide range of epitope types. Nanobody binders specific for membrane proteins have been formulated as therapeutics.^8,9^ They can also find use as tools for structural biology as crystallization chaperones,^10–12^ as bulking fiducials for cryo-EM,^13,14^ and have been applied as modulators of cellular processes by acting on integral membrane protein receptors.^15,16^ In either application, the formation of stable nanobody-membrane protein complexes with dissociation constants (*K*_d_) in the nanomolar to picomolar range are desired.

A variety of techniques are available with which to characterize membrane protein–nanobody complexation and most will provide a measure of the corresponding binding constant. Surface plasmon resonance (SPR),^11^ bio-layer interferometry (BLI),^13^ and isothermal calorimetry (ITC)^12^ have been used for this purpose. Microfluidics approaches wherein protein–ligand binding is tracked by measuring molecular diffusion or hydrodynamic size changes offer advantages in terms of ease-of-use, speed, and sample consumption.^17–25^ Flow-induced dispersion analysis (FIDA) is a relatively new bioanalytical technique which provides a quantitative measure of binding affinity and particle size.^19–25^ FIDA provides the apparent hydrodynamic radius (*R*_h_) of the analyte by measuring its dispersion in solution in a capillary under laminar flow. The measurement is highly efficient requiring just nanograms of protein. For studies of protein–protein interactions, one of the partner proteins is typically fluorescently labelled. This is conveniently carried out with fluorescein isothiocyanate (FITC) or Alexa Fluor 488, both of which are compatible with the 480 nm emission filter of the FIDA instrument detector. To date, FIDA has been used to study soluble protein complexes involving antibodies,^21,22^ nanobodies,^23^ and *de novo* designed miniproteins.^24^

Despite the extensive use made of nanobody binders in the membrane protein field,^7,8,10–14^ FIDA as a means for characterising membrane protein–nanobody complexation has received relatively little attention. More generally, the application of FIDA to standard membrane protein samples where the target is reconstituted in detergent has not been widely demonstrated, despite the proven amenability of FIDA to systems with significant complexity and viscosity.^20,21,24^ A recent relevant application in this regard has been a study of how BacA, a membrane integral undecaprenyl phosphate recycling protein from *enterococci*, interacts with bacteriocin peptides. In this work, BacA, reconstituted in *n*-dodecyl-d-maltoside (DDM) detergent micelles, formed complexes with fluorescently labelled peptide binders. FIDA was conducted using samples in detergent-containing buffer. The resulting *K*_d_ values were in the single digit nanomolar range in line with binding data obtained by ITC analysis.^25^ In the current work, we describe how FIDA can be used to characterise the binding of a nanobody to a detergent-solubilized membrane protein. The membrane protein target is the heterodimeric ATP binding cassette (ABC) transporter complex from the thermophilic bacterium *Thermotoga maritima.*^10,11^ The nanobody binder Nb2 was previously raised against the complex by camelid immunization and shown to bind to the intracellular domain of the transporter (Fig. 1) with low nanomolar affinity, as determined by SPR.^11^ The ABC transporter-Nb2 system is a classic example of one-to-one complexation of a small protein binder (∼19 kDa) with a large membrane protein complex (∼137 kDa). The availability of an ABC transporter-Nb2 co-crystal structure and SPR-derived binding constants renders this system an ideal model with which to evaluate and compare the utility of FIDA for characterising membrane protein–nanobody complexation.

**Fig. 1:**
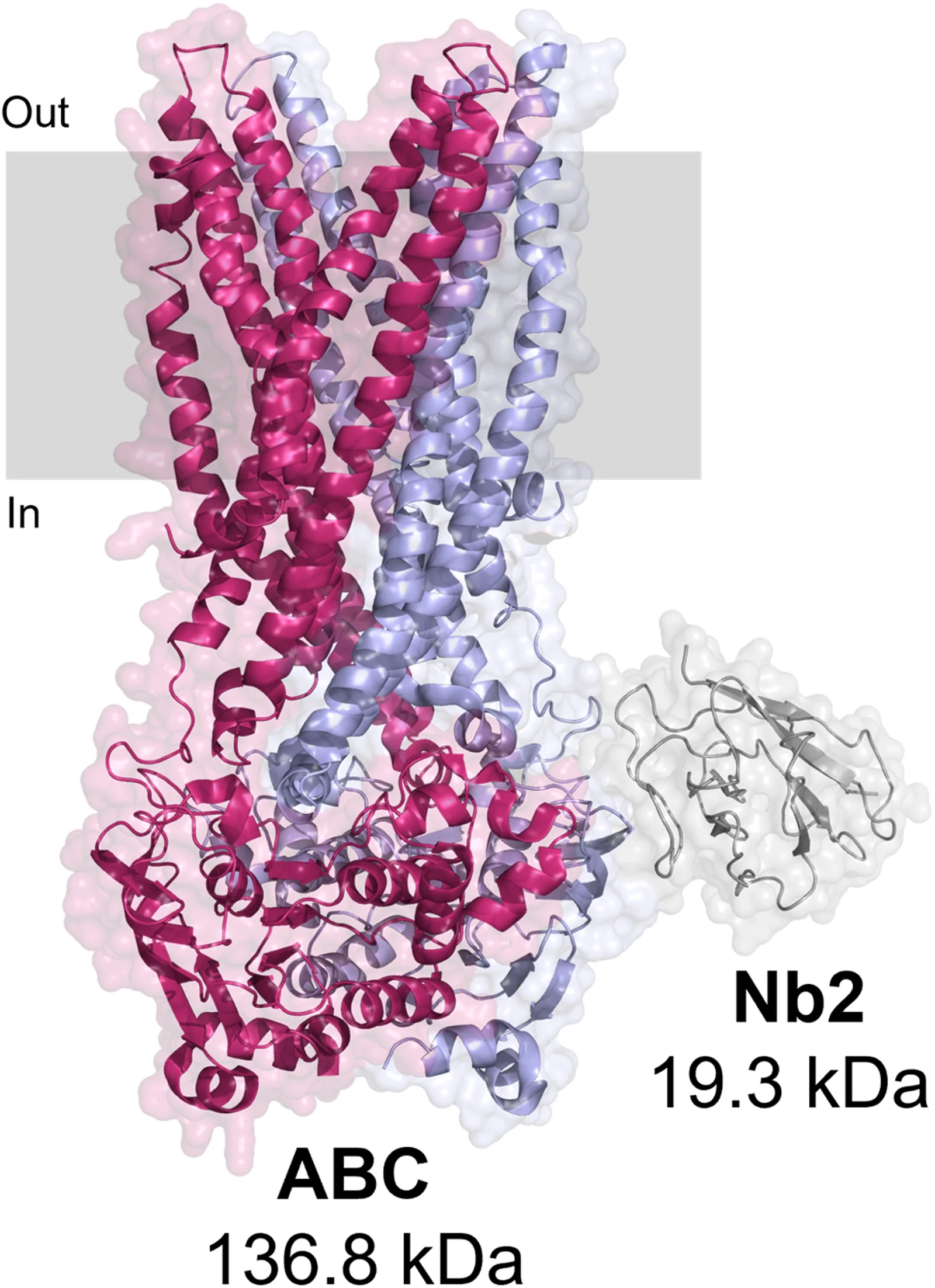
Co-crystal structure of the ABC transporter-Nb2 complex (PDB 6qv2). Approximate membrane boundaries are indicated with the grey background.

The ABC transporter and Nb2 were produced and purified as described previously^7,11^ (Fig. S1 and S2). The corresponding plasmids used in the current work were obtained from the Synthetic Nanobody (Sybody) Generation Toolbox (Addgene Kit #1000000160) developed by Markus Seeger and colleagues^7^ and are identical to those used in the previously described SPR work.^11^ The ABC transporter construct differs from the native protein in that a fifteen residue C-terminal Avi-tag extension is present, previously used to facilitate immobilization onto SPR sensor chips *via* biotin–avidin binding.^11^ In line with the SPR work, the ABC transporter was biotinylated at the Avi-tag in the current study. The Nb2 construct contains an extended C-terminus made up of a ten-residue Myc affinity sequence followed by a flexible linker and a hexahistidine tag (Fig. 2A). Although SPR measurements were carried out with the extended C-terminus on Nb2 in place, crystallization and structure determination of the ABC transporter-Nb2 complex by MX was achieved using the tag-free form of Nb2. An inspection of the ABC transporter-Nb2 co-crystal structure (Fig. 1) revealed the Nb2 C-terminus to be located sufficiently far from the major epitope/paratope binding interfaces to render direct steric impediments to complexation unlikely.

**Fig. 2:**
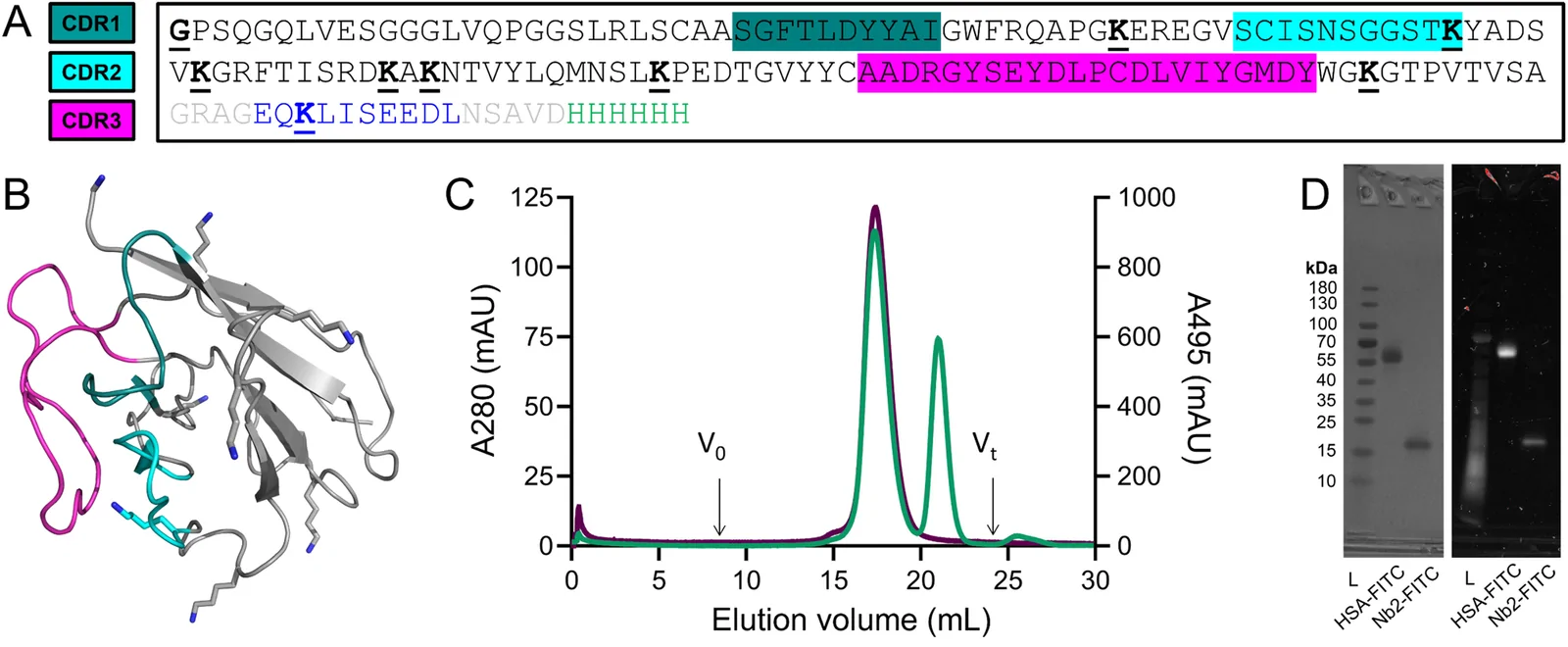
(A) Amino acid sequence of the Nb2 construct used in this work. CDRs are highlighted and differentiated by colour. All possible sites for FITC conjugation are underlined and in bold. Myc-tag, hexahistidine tag, and linker sequences are coloured blue, green, and grey, respectively. (B) View of Nb2 in the ABC transporter-Nb2 co-crystal structure (PDB 6qv2). The orientation of Nb2 corresponds to that in the complex in Fig. 1. CDR regions are coloured as per the sequence in (A). The protein is shown in cartoon representation with lysine side chains shown as sticks. (C) Analytical SEC chromatogram traces collected at 280 nm (purple) and 495 nm (green) for FITC-treated Nb2. The peak with an elution volume of 17.5 mL observed at both 280 nm and 495 nm corresponds to Nb2-F. Free unreacted FITC elutes at 21.5 mL. Column void (*V*_0_) and total (*V*_t_) volumes are 8 mL and 24 mL, respectively. (D) (Left) SDS PAGE (Coomassie stain) of SEC-purified Nb2-F. (Right) Imaging of the same SDS PAGE gel before Coomassie staining using a fluorescein filter. FITC-labelled human serum albumin (HSA-FITC; MW ∼ 66 kDa) is included for reference.

Following the convention that the smaller analyte in FIDA experiments is fluorescently labelled, Nb2 was chosen to be the reporter and was labelled with FITC. The impact that fluorescent labelling might have on Nb2 binding to the ABC transporter was considered by inspecting the Nb2 construct amino acid sequence for lysine residues, which are the primary sites for reaction with FITC. Six lysines were identified in the nanobody itself, with a seventh residing in the Myc tag (Fig. 2A). Of the six sites in Nb2, Lys32 is the only one situated in a complementarity-determining region (CDR), specifically CDR2. Interestingly, CDR2 does not make direct contact with the transporter in the co-crystal structure (Fig. 1 and 2B) and is unlikely to impact binding upon labelling. The remaining lysines and the free amino N-terminal conjugation sites are not proximal to any of the Nb2 CDRs (Fig. 2B) and therefore the probability of FITC labelling compromising ABC-Nb2 binding was deemed low.

Nb2 labelling was carried out in the presence of a molar excess of FITC. The labelled protein was purified from unreacted free dye by a two-step protocol. After the residual reactive FITC was quenched, the reaction mixture was loaded onto a PD-10 desalting column. The free dye which moves slowly through the solid matrix was clearly visible in the column bed (Fig. S3). The resulting fluorescently labelled nanobody (Nb2-F) sample was concentrated and loaded onto an analytical size exclusion chromatography (SEC) column pre-equilibrated with FIDA Buffer (20 mM Tris-HCl pH 7.5, 150 mM NaCl, 1 mM MgCl_2_, 0.03% (w/v) DDM) with the detector set to collect at wavelengths for protein (280 nm) and for fluorescein (495 nm). A Gaussian main peak, indicative of a homogenous Nb2-F protein sample, was evident in the resulting chromatograms (Fig. 2C). A later-eluting peak was detected at 495 nm and was deemed to be the unconjugated FITC that had not been removed in the previous purification step. A comparison of the main peak signals at 280 nm and 495 nm indicated a labelling ratio of approximately four fluoresceins per Nb2 molecule (Calculation S1). Subsequent SDS PAGE analysis of the Nb2-F sample post-SEC showed that the protein was pure and free of unreacted FITC as evidenced by the absence of a signal at the dye front when viewed through a fluorescein filter (Fig. 2D).

The underlying principle on which FIDA is based is that the *R*_h_ of a particle determines its dispersion in solution under conditions of laminar flow in a thin capillary.^17–19^ When the particle is intrinsically fluorescent or has been fluorescently labelled, dispersion can be conveniently measured by recording the fluorescent profile in the solution in flow as a function of time. Binding to a partner will usually bring about a change in *R*_h_ which can be monitored by a change in the dispersion profile (Fig. 3A). Repeating the profile measurement at increasing concentrations of the partner can be used to generate a binding isotherm from which a binding constant can be derived.

**Fig. 3:**
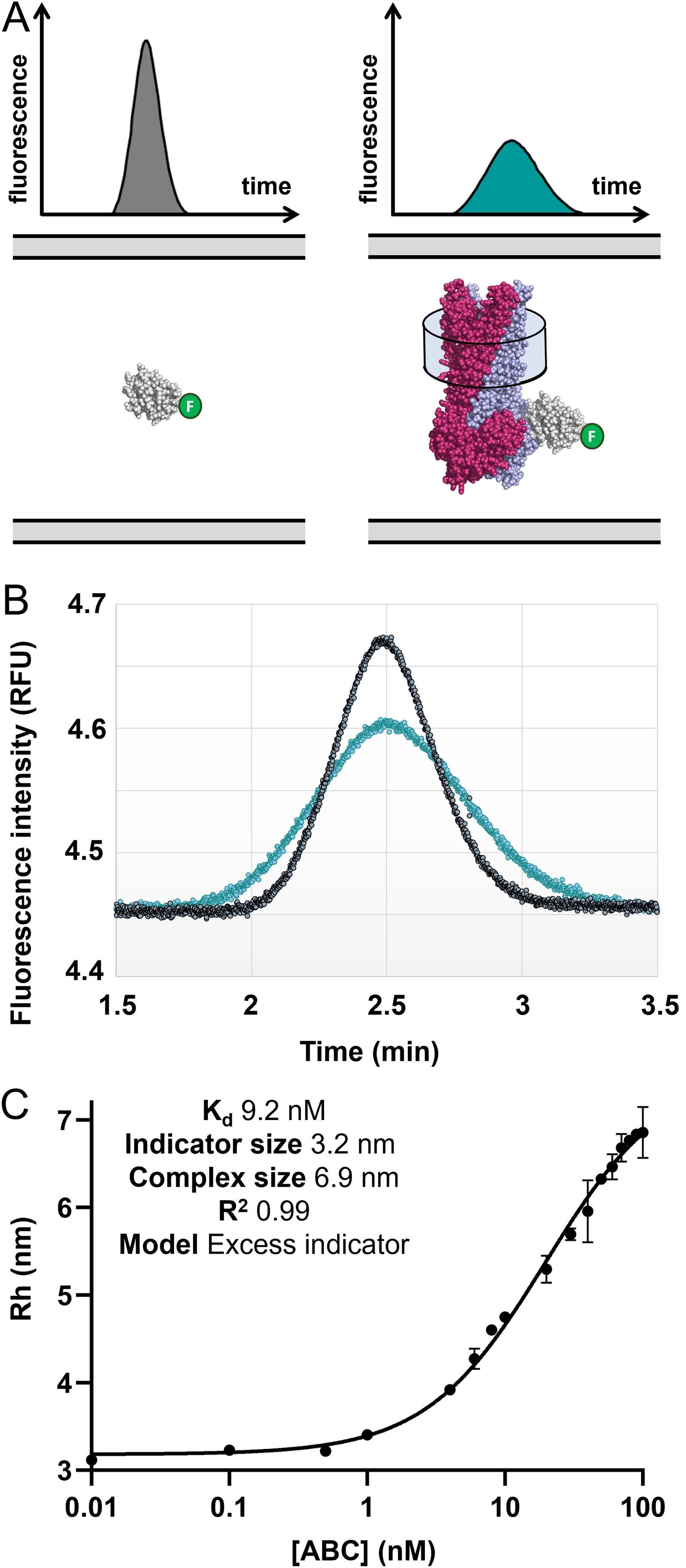
(A) Schematic illustrating changes in the dispersion profiles of Nb2-F upon binding to the ABC transporter during a FIDA experiment. Proteins are shown as space-filling models and coloured as in Fig. 1. ABC is depicted reconstituted into a DDM micelle, represented as a blue cylinder. Capillary boundaries are shown as horizontal bars. (B) Representative dispersion profiles recorded for samples containing 0 (black) and 100 (teal) nM ABC transporter. (C) Binding isotherm derived from FIDA runs at different ABC transporter concentrations. Experiments were run in triplicate, and standard deviations are indicated by error bars. Indicator and complex size refer to the *R*_h_ measured at the beginning (0 nM ABC) and end (100 nM ABC) of the titration.

Initial FIDA runs with Nb2-F alone in FIDA buffer at 10 °C gave an *R*_h_ value of 3.2 nm. This matched the *R*_h_ value of ∼3 nm calculated using HullRad, an algorithm for predicting hydrodynamic properties of macromolecules from structure files. HullRad estimates *R*_h_ values reliably within ∼5% of experimental values.^26^ The structure file used in this case was the Nb2 model from the ABC-Nb2 co-crystal structure (PDB 6qv2, Fig. 1 and 2B).

Binding isotherm data collection on the transporter/Nb2-F system began by pre-equilibrating the capillary with unlabelled ABC transporter protein solution. Subsequently, premixed solutions containing 50 nM Nb2-F and 0 to 100 nM ABC transporter protein were introduced into the capillary under flow with each subjected to dispersion profiling (Fig. 3B and S4). The FIDA data were processed, and *R*_h_ values (Table S1) were plotted *versus* ABC transporter concentration (Fig. 3C). The *R*_h_ value recorded for an equimolar mix of ABC transporter and Nb2-F was 6.3 nm which aligns well with the value of 6.2 nm estimated for the ABC-Nb2 complex structure (PDB 6qv2) using HullRad. An *R*_h_ value of 6.9 nm was recorded at the highest concentration of ABC transporter of 100 nM used in this study.

The binding isotherm was initially fitted to a 1 : 1 binding model yielding a *K*_d_ of 8.4 nM (Fig. S5). This 1 : 1 binding model is the most common approach employed in fitting FIDA data for protein–ligand systems.^23–25^ However, this binding model does not result in best fits for FIDA-derived isotherms where the binding affinity between the indicator and analyte is high *i.e.*, in the single digit nanomolar to picomolar range. As described by Pedersen *et al.*,^21^ and predicted in earlier work,^20^ in such tight binding systems, the indicator concentration cannot be assumed to be less than the *K*_d_ and therefore the calculated affinity may be skewed and inflated. The underlying problem with such systems is that, when making use of Langmuir isotherms, one component of the system must be in vast excess over the other so that binding does not cause depletion of one of the species.^27^ This necessitates the use of an ‘excess indicator binding isotherm’,^21,22^ a binding model which makes use of a quadratic equation to account for both the total indicator and total analyte concentrations explicitly and is available within the FidaBio software package. The need to make use of the excess indicator binding model was identified after receiving comments regarding the fit of the isotherm (Fig. S5) during the peer review process. Fitting with the excess indicator model yielded a modest visual improvement of the fit (Fig. 3C) and a slight change in the calculated *K*_d_ (from 8.4 nM to 9.2 nM). Interestingly, the divergence from an ideal sigmoidal isotherm shape observed in Fig. S5 becomes apparent as the concentration of membrane protein analyte is increased above the single digit nanomolar *K*_d_ values determined both by SPR and FIDA. In comparison, FIDA-derived binding isotherms for BacA-peptide complexation appear to exhibit better fits with the 1 : 1 binding model at 0 to 100 nM membrane protein.^25^ Compared to the ABC-Nb2 system, however, *K*_d_ values for the BacA system are significantly higher (∼100 nM) and therefore the same issues regarding indicator concentration should not apply.

SPR and FIDA measurements have now been used to characterise the binding of Nb2 to the ABC transporter. Both arrive at binding constants in the single digit nanomolar range, ∼1 nM by SPR^11^ and ∼9 nM by FIDA (Fig. 3C). SPR measurements investigated the complexation of this system in the context of the conformational state of the receptor with and without adenosine triphosphate (ATP) bound. Although ATP induces significant structural rearrangement of the ABC transporter, negligible differences in Nb2 affinity were observed with *K*_d_ values of 0.8 and 0.93 nM reported for the free and ATP-bound states, respectively.^11^ The present FIDA experiments were conducted with the ATP-free form of the receptor using identical buffer conditions as in the SPR experiments.

The SPR experiments performed with ABC and Nb2 involved immobilising the biotinylated ABC transporter onto avidin-coated sensor chips followed by the measurement of an SPR signal in response to a flowing solution containing nanobody over a range of concentrations up to 73 nM Nb2. In these experiments, the effective concentration of the ABC transporter on the sensor chip surface was estimated at ∼150 μM. In the FIDA study, neither binding partner was surface-tethered. Rather, solutions containing 50 nM Nb2-F and from 0 nM to 100 nM ABC transporter were passed through a capillary and dispersion was quantified under laminar flow conditions (Fig. 3A). Despite the differences in the nature of the two methods, both provide data in support of the conclusion that Nb2 binds to the ABC transporter with single digit nanomolar affinity which corresponds to tight binding.^28^ Relatedly, in the BacA study referred to earlier, this small membrane phosphatase was found to bind to a toxic bacteriocin peptide with an affinity of ∼100 nM as measured by FIDA. The corresponding value obtained by ITC was still in the nM range but three times higher.^25^

Biophysical methods not requiring immobilization such as FIDA and ITC offer significant advantages over approaches that require immobilization of one of the interacting partners, such as SPR or BLI.^29,30^ Use of non-immobilization methods which measure binding in solution generally reduces the risk that surface attachment, steric constraints, or altered membrane protein conformation will influence binding behaviour.^31^ This is particularly valuable for membrane proteins, where maintaining the native or solution-state conformation can be critical for obtaining physiologically relevant affinity measurements. In terms of time and sample consumption, FIDA can provide rapid determination of binding affinity while requiring relatively small sample quantities and is amenable to higher-throughput measurements.^20–25^ ITC measurements are generally slower and require significantly more sample, while providing a more comprehensive thermodynamic characterization including binding affinity, stoichiometry, and binding enthalpy.^25,29,30^ In comparison, immobilization-based platforms can require substantial assay development and surface-functionalization steps, and consumables costs can become significant when large numbers of samples are screened. Non-immobilization methods such as FIDA and ITC therefore have the potential to reduce overall assay costs by eliminating immobilization reagents and reducing sample requirements, while also simplifying workflows and enabling direct analysis of interactions in solution.

Despite its advantages as an immobilization-free, low-volume approach, FIDA has several limitations that should be considered when characterizing ligand binding to membrane protein targets. Most importantly, the experimentally accessible affinity range is constrained by the solution concentrations of ligand and membrane protein that can be prepared reliably. For weak interactions, accurate determination of binding constants generally requires ligand concentrations substantially above the expected *K*_d_.^27^ However, membrane–protein ligands may have limited aqueous solubility, aggregate at higher concentrations, or require detergents, lipids or other membrane mimetics that themselves can restrict the usable concentration range. Consequently, weak membrane protein–ligand interactions may be difficult to characterize quantitatively if sufficiently high ligand concentrations cannot be reached. Fortunately, nanobodies are noted for their high aqueous solubility. FIDA is also fundamentally dependent on detecting a measurable change in the hydrodynamic radius of the fluorescent indicator upon complex formation, meaning that interactions involving a relatively small ligand or a large membrane protein target may produce a small change in apparent size and therefore reduced sensitivity. Recent studies have specifically identified this dependence on size change as a limitation for protein–small molecule interactions and for larger protein targets.^32^ For our model ABC-Nb2 system, apparent particle size more than doubled on complex formation (Fig. 3). In addition, membrane proteins present particular practical challenges in relation to their solubilization state, detergent/lipid environment, and oligomeric state which can influence both hydrodynamic size and binding behaviour,^33,34^ potentially complicating interpretation of dispersion measurements. Non-specific adsorption to the capillary can also occur and may require surface coating and optimization of assay conditions.^35^

Fortunately, FITC labelling of Nb2 did not appear to impede complexation with the ABC transporter despite the relatively large size of the fluorescein dye (∼340 Da), the small size of Nb2 (∼19 kDa), and conjugation occurring at multiple sites on the protein (Calculation S1). Nb2 conforms to the general trend where nanobodies most commonly associate with cognate antigens *via* CDR3 (Fig. 1 and 2B). The absence of FITC-reactive lysines in CDR3 of Nb2 was fortuitous in this respect. Interestingly, lysine is among the least frequently occurring amino acids found in CDR3 of nanobodies and is even rarer in CDR1 and CDR2 sequences.^36^ We do not therefore anticipate labelling of nanobodies with excess FITC, as carried out here, to negatively impact their binding to membrane protein targets in the majority of cases. Furthermore, optimization of reaction conditions to limit the extent of labelling and to minimise the impact on binding, while always an option, may not be necessary.

## Conclusions

FIDA is an exciting new bioanalytical technique for measuring the binding affinity of macromolecules in solution and for hydrodynamic radius determination using very small amounts of analyte. While FIDA has been applied to a wide range of soluble proteins and protein complex types,^20–25^ the application of FIDA to membrane proteins has been underexplored to date. With this study, we demonstrate the utility of FIDA as a fast and simple method for characterising the association of a membrane protein in a detergent micelle with a soluble protein binder. The technique is efficient in terms of sample consumption (micrograms of protein in nanolitre volumes required for full ligand titrations) and speed of setup and data collection (experiments are on the order of minutes). FIDA is non-destructive and does not require immobilization, therefore it is feasible that samples may be reused. For example, post-FIDA protein–protein complexes may be prepared on grids and structurally characterised by cryo-EM.^3,4,13,14^

Measurements were conducted in a standard detergent-containing buffer solution akin to buffers used in many membrane protein studies. The Fida1 system used in this study was amenable to characterising the ABC transporter-Nb2 system and recapitulated the single digit nanomolar binding affinity obtained previously by SPR.^11^ FIDA is advantageous to other biophysical techniques in use for characterising protein–protein complexation in that binding quantitation is carried out alongside particle sizing (Fig. 3C). Using FIDA, it was possible to define the hydrodynamic radius of our membrane protein–nanobody complex. We anticipate this feature of FIDA to be particularly useful for the detection of nanobody self-association and of binding at multiple sites providing useful quality control and binding stoichiometric information. The current study and other previously referenced work^25^ show that FIDA can be used with membrane proteins solubilized in maltose-based detergent micelles. Such non-ionic detergents, that include DDM, are extensively used in membrane protein structural biology and appear well-suited for carrying out FIDA to evaluate target quality, stability, and suitability as a prelude to downstream structure and function work.

We have highlighted above our process of selecting the most appropriate binding model for fitting of our isotherm (Fig. 3C and S5) with a view to guiding prospective new users interested in applying FIDA to characterising membrane protein–nanobody complexation. While use of the excess indicator model did not alter our isotherm fit and resulting *K*_d_ as drastically as has been observed with other systems,^21^ the selection of the most appropriate model to fit FIDA data remains a critical step in making reliable binding affinity determinations. We posit that the excess indicator model will constitute the best model to use for fitting of FIDA data for most membrane protein–nanobody systems given that tight binding is usually selected for in the nanobody discovery process.^5,7^

The FIDA method implemented in this study made use of fluorescence to monitor the diffusional characteristics of a fluorescently labelled nanobody with and without a binding partner, the ABC transporter protein, under conditions of laminar flow. The nanobody was conveniently labelled using a high concentration of FITC. The product, labelled with approximately four molecules of fluorescein per Nb2 molecule, was purified from excess unreacted FITC using a small desalting column followed by analytical SEC. The major peak fractions in the size-exclusion chromatogram were concentrated, shown to be free of excess FITC, and used directly for FIDA. We expect this simple labelling and purification protocol to be applicable to future nanobody binding projects. Based on the limited results available to date, FIDA appears to be as efficient a method for characterising the complexation of membrane proteins and nanobody binders as the more established techniques such as SPR, ITC, and BLI. The FIDA technique is likely to prove useful for screening panels of nanobodies for high-affinity binders in high-throughput fashion using miniscule amounts of protein.

## Author contributions

K. O. R., M. C., and A. Q. conceived the project, guided analyses, and wrote the manuscript; K. O. R. and S. G. M. produced the membrane protein and nanobody samples; M. O. performed the FIDA experiments and processed the data; all authors contributed to the interpretation of the data and commented on the final manuscript.

## Conflicts of interest

There are no conflicts to declare.

## Supplementary Material

AN-151-D6AN01036A-s001

## Data Availability

The data supporting the findings of this study are available within the article or its supplementary information (SI). Supplementary information is available. See DOI: https://doi.org/10.1039/d6an01036a.
