# Supplementary material for: Characterization of membrane protein–nanobody complexation via flow-induced dispersion analysis (FIDA)
Source: Analyst. 2026 Oct 29;151(22):6661–7. doi: 10.1039/d6an01036a (PMC13725375; doi:10.1039/d6an01036a)
Supplement: Supplementary file 1 [file AN-151-D6AN01036A-s001.pdf]

Supplementary Information (SI) for Analyst.
This journal is © The Royal Society of Chemistry

SUPPORTING INFORMATION

Characterization of Membrane Protein-Nanobody Complexation via Flow-Induced Dispersion Analysis (FIDA)

Kiefer O. Ramberg,[a,b,*] Monika Olejnik,[c] Saúl Gallego-Martínez,[b] Andrew Quigley,[d] and Martin Caffrey[b,*]

[a]School of Chemical Sciences, Dublin City University, Glasnevin, Dublin 9, D09 V209, Ireland.

[b]School of Biochemistry & Immunology, Trinity College Dublin, Dublin, D02 R590, Ireland.

[c]Astbury Centre for Structural Molecular Biology, School of Molecular and Cellular Biology, Faculty of Biological Sciences, University of Leeds, UK.

[d]Membrane Protein Laboratory, Diamond Light Source, Research Complex at Harwell, Didcot, UK.

*Correspondence: kiefer.ramberg@dcu.ie and martin.caffrey@tcd.ie

Contents

	Experimental	3
**Fig. S1**	Production and purification of the ABC transporter protein	7
**Fig. S2**	Production and purification of the Nb2 protein	8
**Fig. S3**	PD-10 column post elution of FITC-labelled Nb2 (Nb2-F)	9
**Calculation S1**	Quantitation of moles of FITC conjugated per mole of Nb2-F	10
**Fig. S4**	Representative dispersion profiles	11
**Table S1**	Hydrodynamic radius calculated from FIDA data	12
**Fig. S4**	Binding isotherm with fitting to a 1:1 binding model	13
**References**		14

Experimental

Production and purification of the ABC transporter. The pBXNH3LCA_TM287/288 plasmid – 50 ng (amp^r) was obtained from the Synthetic Nanobody (Sybody) Generation Toolbox (Addgene Kit #1000000160)[1] and transformed into C43 (DE3) cells overnight (~16 h). The next day a single colony was used to inoculate 100 mL of Luria-Bertani (LB) medium supplemented with ampicillin (100 mg/L) which was incubated overnight (16 h) at 30 °C and 180 rpm. The next day, 10 mL aliquots of the starter culture were used to inoculate three 3 L baffled flasks, each containing 1 L of Terrific Broth (TB) supplemented with ampicillin (100 mg/L), which were incubated at 37 °C and at 180 rpm. Once the culture had reached an optical density at 600 nm (OD600) of 1.4 (~4 h), expression was induced by adding L-arabinose to a final concentration of 0.016 %(w/v). The cultures were incubated for 5 h at 30 °C with shaking at 180 rpm. The cells were harvested by centrifugation for 10 min at 6,000 x *g* and 4 °C. The resulting 19 g of wet cell pellet was flash-frozen in liquid nitrogen and stored at -70 °C.

All subsequent steps were carried out at 4 °C unless otherwise noted. The cell pellet was thawed at room temperature (RT, 20 - 22 °C) and resuspended in 38 mL **Buffer 1** (20 mM Tris-HCl pH 7.5 (at RT), 200 mM NaCl, 10 %(v/v) glycerol) supplemented with 5 mM $MgCl_2$ and 0.02 mg/mL DNAse. After straining the cell suspension to remove clumps, the cells were lysed by passage five times through an Emulsiflex C5 cell disruptor at 15,000 psi. The cell lysate was centrifuged at 25,000 x *g* for 30 min. The supernatant was centrifuged at 150,000 x *g* for 2 h and the resulting 4.8 g of membrane pellet was resuspended in **Buffer 1** using a Dounce homogeniser. Resuspension used 8 mL of **Buffer 1** for every 1 g of membrane pellet. Solubilisation was carried out by adding n-dodecyl-D-maltoside (DDM) detergent to the resuspended membrane pellet to a final concentration of 1.5 %(w/v) with mixing on a wheel mixer overnight (18 h) at 12 rpm. Insoluble material was removed by centrifugation at 150,000 x *g* for 1 h. The supernatant, containing solubilized membrane proteins, was supplemented with 20 mM imidazole. 3 mL Ni-NTA resin (Cube Biotech Cat # 31103), pre-washed with 20 mL of **Buffer 2** (50 mM imidazole-HCl pH 7.5 (at RT), 200 mM NaCl, 10 %(v/v) glycerol, 0.03 %(w/v) DDM), was added and the mixture was incubated on a wheel mixer at 12 rpm for 1 h. The mixture was loaded onto a gravity column and washed with 60 mL (20 column volumes) of **Buffer 2**. The bound histidine-tagged TM287/288 ABC transporter complex was eluted with **Buffer 3** (200 mM imidazole-HCl pH 7.5 (at RT), 200 mM NaCl, 10 %(v/v) glycerol, 0.03 %(w/v) DDM). Eluted fractions were pooled (~21 mL) and exchanged into **Buffer 4** (20 mM imidazole-HCl pH 7.5 (at RT), 200 mM NaCl, 10 %(v/v) glycerol, 10 mM magnesium acetate, 0.03 %(w/v) DDM) using a PD-10 column (Cytiva). The resulting ~28 mL sample contained 12 mg protein (0.43 mg/mL, estimated using an extinction coefficient of 86,750 $M^{-1}.cm^{-1}$ for the ABC transporter calculated in ProtParam).[2]

Biotinylation of the Avi-tag on the C-terminus of the ABC transporter TM288 chain was performed by the addition of the biotin ligase enzyme, BirA (produced in-house bearing a hexahistidine tag), and a two-fold molar excess of biotin as described.[1] To remove the N-terminal decahistidine tag at the N-terminus of the TM287 chain, HRV-3C Protease (SIGMA Catalogue #SAE0045) was added at a 1:10 HRV-3C protease:ABC transporter by weight ratio. Biotinylation and cleavage of the His tag was allowed to proceed simultaneously overnight (18 h) with gentle rocking. The next day, a reverse IMAC step was performed to remove the His-tagged BirA and HRV-3C protease. 3 mL Ni-NTA resin (Cube Biotech Cat # 31103) pre-washed with 20 mL **Buffer 4** was added and the mixture was incubated on a wheel mixer at 12 rpm for 1 h. The mixture was loaded onto a gravity column, and the protein solution was subjected to a total of 3 passes over the resin. The ABC transporter was further purified by size exclusion chromatography (SEC) using a Superose 6 increase 10/300 GL column equilibrated with **FIDA buffer** (20 mM Tris-HCl pH 7.5 (at RT), 150 mM NaCl, 1 mM $MgCl_2$, 0.03 % (w/v) DDM). The peak fractions were concentrated using a Millipore centrifuge filter with a molecular weight cut-off of 100 kDa, snap frozen in liquid nitrogen, and stored at -70 °C until required. Purified stocks were analysed by SDS PAGE and Coomassie staining (**Figure S1**). Protein concentration was determined spectrophotometrically at 280 nm using an extinction coefficient of 86,750 M^{-1} cm^{-1} determined in ProtParam,[2] as described above.

Production and purification of Nb2. Nb2 was produced and purified as described.[3] The plasmid for Nb2 was obtained from the Synthetic Nanobody (Sybody) Generation Toolbox (Addgene Kit #1000000160)[1] and comprised the Nb2 sequence subcloned into the pSb_init expression vector (Addgene #110100). The Nb2 plasmid – 50 ng (amp^r) was transformed into C43 (DE3) cells overnight. The next day a single colony was used to inoculate 50 mL of LB supplemented with ampicillin (100 mg/L) which was incubated overnight (16 h) at 37 °C and 180 rpm. The next day, 10 mL aliquots of the starter culture were used to inoculate two 3 L baffled flasks containing 1 L of TB supplemented with ampicillin (100 mg/L) which were then incubated at 37 °C and at 180 rpm. Once the culture had reached an OD600 of 0.5, the temperature was reduced to 22 °C and the culture was incubated for a further 1.5 h at 180 rpm. Expression was induced by adding L-(+)-arabinose to a final concentration of 0.02 %(w/v). The cultures were incubated overnight (16 h) at 22 °C with shaking at 180 rpm. Cells were harvested by centrifugation for 10 min at 6,000 x *g*. The resulting 11 g combined wet cell pellet was flash-frozen in liquid nitrogen and stored at -70 °C.

Purification began by thawing the cell pellet at RT before resuspending the cells in 50 mL of **TBS** (50 mM Tris-HCl pH 7.5 (at RT), 150 mM NaCl). $MgCl_2$ was added to a final concentration of 2 mM and DNase I was added to a final concentration of 25 µg/mL. The cell suspension was incubated on ice for

30 min. The cells were lysed in a high-pressure homogeniser (3 passes at 15,000 psi), and cell debris and intact cells were removed by centrifuging at 20,000 x *g* for 20 min. The pellet was discarded and imidazole was added to the supernatant to a final concentration of 15 mM. 2 mL Ni-NTA resin (Cube Biotech Cat # 31103), pre-washed with 20 mL of **TBS**, was added and the sample was incubated for 1 h. The sample was then loaded onto a gravity column and the flowthrough was collected. The resin was washed with 25 mL of **Washing Buffer** (TBS supplemented with 15 mM imidazole). The resin was washed with an imidazole gradient (4 mL washes each of **TBS** supplemented with 60, 80, 100, 200, 300, and 500 mM imidazole). The bulk of the Nb2 protein eluted at 80 – 200 mM imidazole and these fractions were pooled and concentrated to ~1.5 mL using a Millipore centrifuge filter with a molecular weight cut-off of 10 kDa. The concentrated fractions were loaded onto a HiLoad 16/60 Superdex 200 SEC column equilibrated with **TBS** (**Figure S2**). Protein purity was evaluated using SDS PAGE with Coomassie staining (**Figure S2**). Protein concentration was determined spectrophotometrically at 280 nm using the extinction coefficient ε = 27,640 $M^{-1}.cm^{-1}$, obtained from the protein sequence using ProtParam.[2] Aliquots (20 µL) of purified Nb2 were snap frozen in 0.5 mL eppendorfs in liquid nitrogen, and stored at -70 °C until required.

Preparation of FITC-labelled Nb2 (Nb2-F). 40 µL of 1 mg/mL FITC (SIGMA F7250) in neat DMSO was added to five 360 µL samples of Nb2 diluted to 1 mg/mL with **Carbonate Buffer** (0.1 M sodium carbonate, pH 9.0 (at RT)). Tubes were wrapped in aluminium foil to protect the samples from light. The FITC labelling reaction was left to proceed at 4 °C overnight (16 h). The next day, the reaction was quenched by adding ammonium chloride to a final concentration of 50 mM. Quenching proceeded on ice for 1 h after which the samples were exchanged into **FIDA buffer** using a PD-10 desalting column (Cytiva) to remove the bulk of the unconjugated FITC. The samples were combined and were then concentrated to ~1 mL using a Millipore centrifuge filter with a molecular weight cut-off of 10 kDa and loaded onto an Superdex 18/60 analytical SEC column pre-equilibrated with **FIDA buffer**. Elution was monitored at 280 nm and 495 nm. Main peak fractions exhibiting absorbance at both 280 nm and 495 nm were pooled (~4 mL). Nb2-F concentration was determined spectrophotometrically at 280 nm using the same extinction coefficient as described above for the unlabelled protein. Aliquots of the Nb2-F protein solution were flash frozen in liquid nitrogen and stored at -70 °C until required.

Flow-induced dispersion analysis (FIDA). Characterisation of the complexation of Nb2-F with the ABC transporter was carried out on a Fida 1 instrument equipped with a 480 nm fluorescence detector and a standard uncoated fused silica capillary with an internal diameter of 75 µm (Fidabio). Experiments were performed with the sample tray and capillary chamber temperature set to 10 °C. Sample

viscosity was set to 0.0013 Pa·s, the dynamic viscosity of liquid water at 10 °C.[4] Prior to sample injection, the capillary was washed and equilibrated with **FIDA buffer** over a period of 20 s. To facilitate reaching binding equilibrium prior to measurement, the "premix" FIDA assay protocol[4] was employed in which Nb2-F and the ABC transporter in **FIDA buffer** were combined in vials and incubated at 10 °C for 10 min before being injected into the Fida 1 instrument. The final concentrations of Nb2-F and ABC in the premixed samples were 50 nM and 0 to 100 nM, respectively. In this method, the free analyte (ABC transporter) at the desired concentration (0 to 100 nM) was injected into the capillary before and after injection of the premixed samples. The pre-mixed samples of Nb2-F and ABC transporter were injected over a period of 10 s, and the complexes were measured under flow over 4 min. This protocol provided a constant analyte concentration in the dispersion zone allowing for the designation of a background, i.e., the signal observed prior to the introduction of the indicator (Nb2-F). All measurements were carried out in triplicate. The FIDA Software Suite was used to calculate the apparent particle hydrodynamic radius (Rh) from each of the resulting dispersion profiles (Taylorgrams). The determined Rh values plotted as a function of ABC transporter concentration were fit to both the 1:1 and excess indicator binding models available as part of the FidaBio software package.[4,5,6]

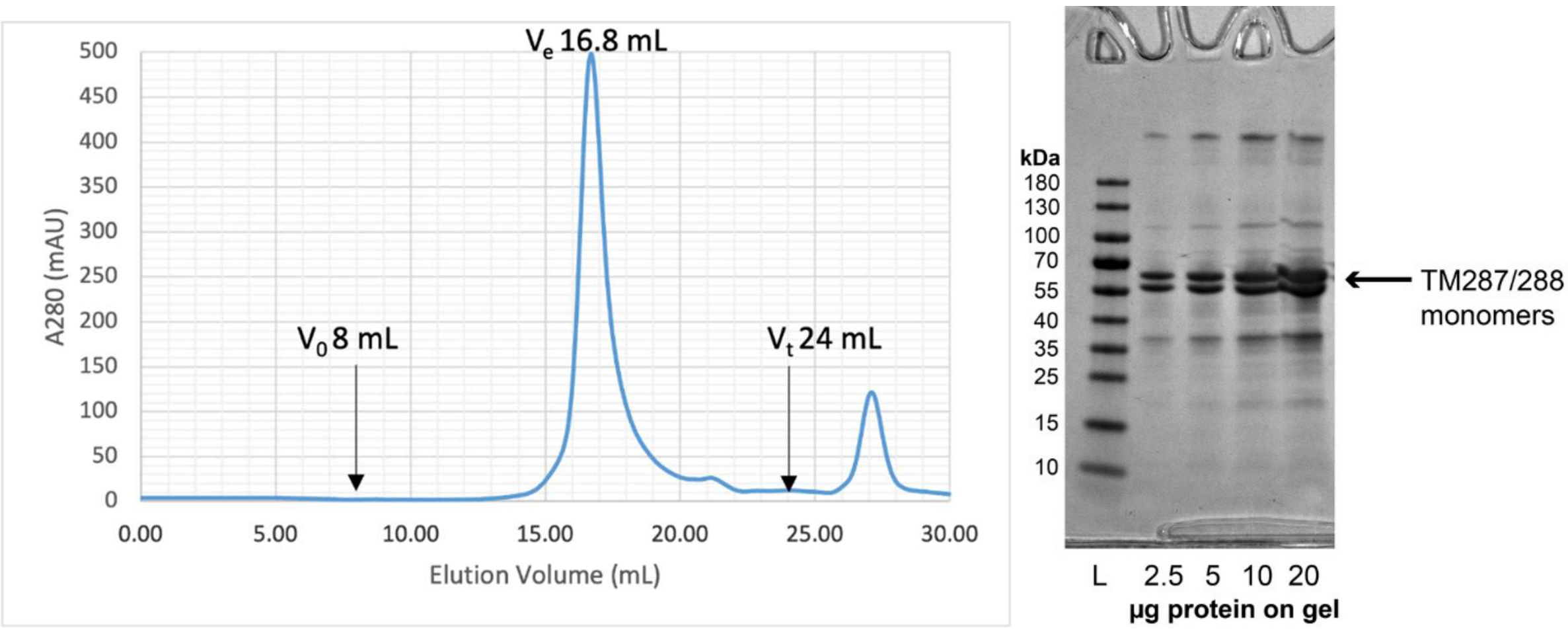

Fig. S1. (*Left*) Gel filtration (SEC) profile of the ABC transporter on a Superose 6 increase 10/300 gl column. The ABC transporter main peak elution volume (V_e) was 16.8 mL. Column void (V_0) and total (V_t) volumes are indicated. **(*Right*)** SDS PAGE (Coomassie stain) loading series of purified ABC transporter. Unheated samples were run on a Biorad SDS PAGE gel with a 4-20 %(w/v) polyacrylamide gradient at 180 V for 40 min at RT.

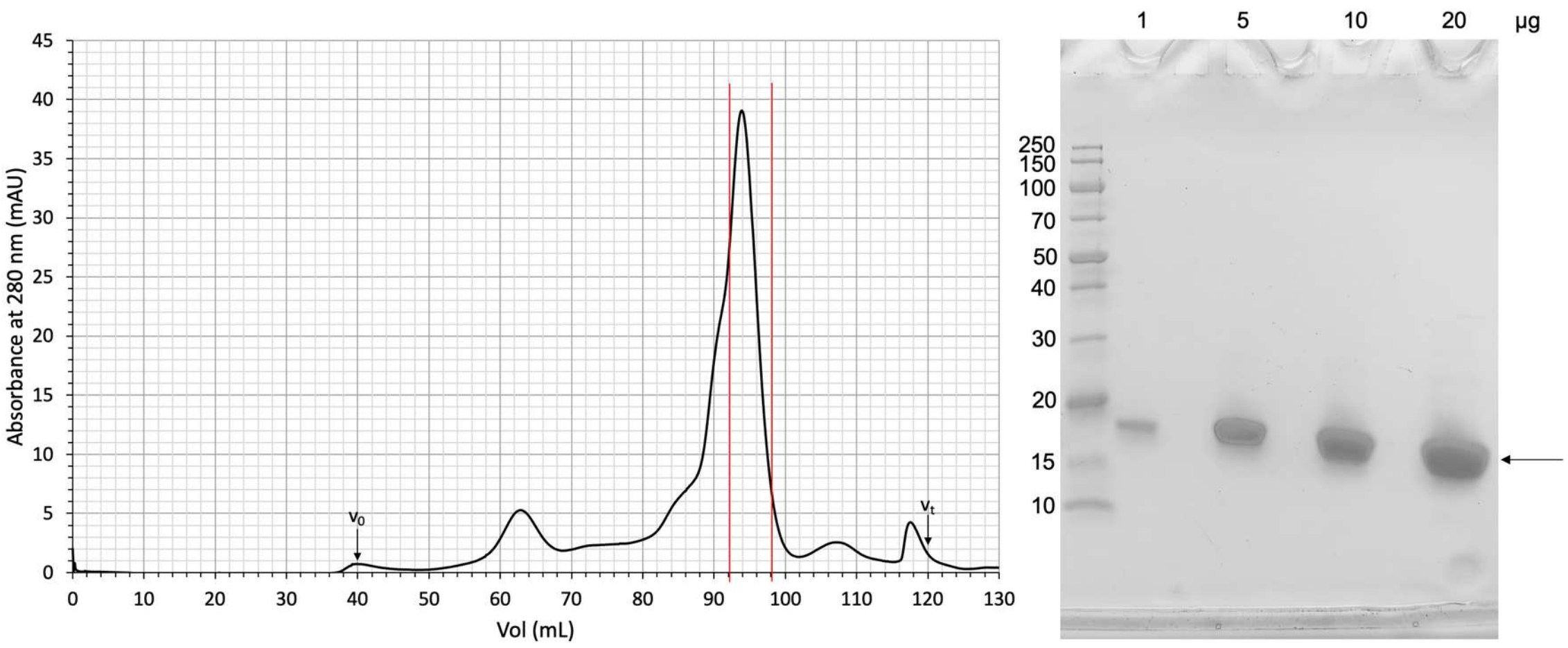

Fig. S2. (*Left*) Gel filtration (SEC) profile of Nb2 on a Superdex 16/60 S200 column. The Nb2 main peak has an elution volume (V_e) of 94 mL. Column void (V_0) and total (V_t) volumes, as indicated on the chromatogram, are 40 and 120 mL, respectively. Red lines indicate the fractions that were combined to give the final Nb2 stock. **(*Right*)** SDS PAGE (Coomassie stain) loading series of purified Nb2. Samples were run on a Biorad 12 %(w/v) polyacrylamide SDS PAGE gel at 180 V for 50 min at RT. Samples were heated to 95 °C for 3 min prior to loading.

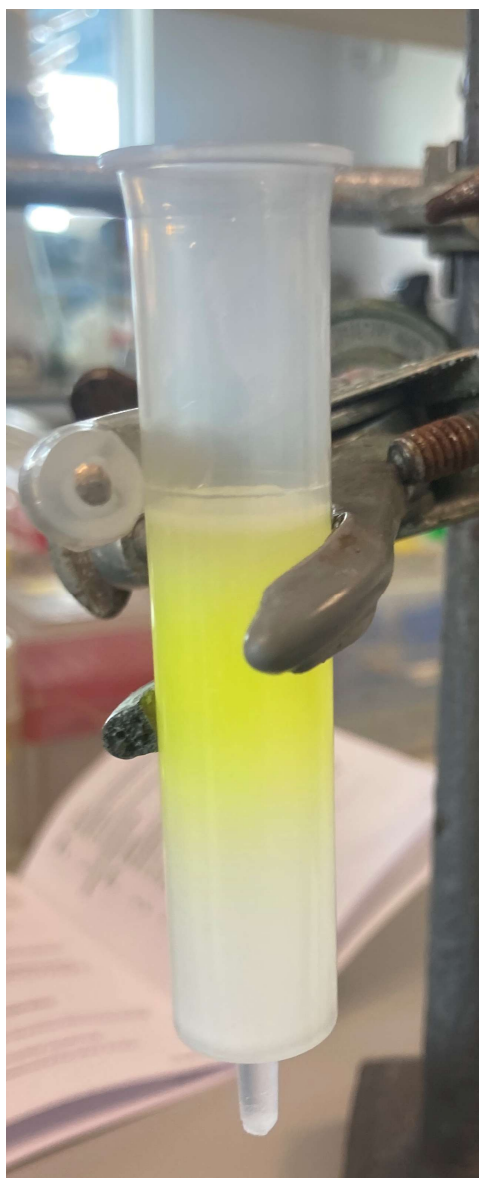

Fig. S3. PD-10 column after the FITC-labelled Nb2 (Nb2-F) was eluted. The unconjugated free dye is evident in the column bed as a diffuse yellow band.

The question

How many moles of FITC are conjugated per mole Nb2 in our stock of Nb2-F?

Given

Molecular weight of Nb2, 19,300 $g.mol^{-1}$

Nb2 molar extinction coefficient at 280 nm (ε), 25,900 $M^{-1}.cm^{-1}$

Molecular weight of FITC, 389 $g.mol^{-1}$

FITC molar extinction coefficient at 495 nm (ε'), 73,000 $M^{-1}.cm^{-1}$

Correction factor (CF_{280}) for FITC contribution to absorbance at 280 nm, 0.35[6]

Assumptions

- The Nb2-F stock is devoid of free dye
- The Nb2 does not absorb at 495 nm

The solution

The concentration of Nb2 (C_{Nb2}) in a diluted sample (dilution factor (DF) = 1,000) of our Nb2-F stock may be determined by measuring absorbance at 280 nm (A_{280}) in a cuvette with a pathlength (l) of 1 cm and using a rearrangement of the Beer-Lambert Law ($A_{280} = \varepsilon \times l \times C_{Nb2}$):

$$C_{Nb2} = (A_{280} / (\varepsilon \times l)) \times DF$$

However, the fluorescein conjugated to Nb2 also contributes to the measured absorbance of Nb2-F at 280 nm.[6] A correction factor (CF_{280}) must therefore be applied to adjust for dye absorbance at 280 nm. To this end, the absorbance of the Nb2-F conjugate at 495 nm (A_{495}) is multiplied by CF_{280} and the product is subtracted from A_{280}. The net value is used in the Beer-Lambert equation to calculate Nb2 concentration in the Nb2-F sample as follows:

$$C_{Nb2} = (A^{net}_{280} / (\varepsilon \times l)) \times DF = (A_{280} - (A_{495} \times 0.35) / (\varepsilon \times l)) \times DF$$

$$= (0.124\ AU - (0.275\ AU \times 0.35) / (25{,}900\ M^{-1}.cm^{-1} \times 1\ cm)) \times 1{,}000$$

$$= 0.001\ M$$

The concentration of dye (C_{FITC}) in the sample of the Nb2-F stock is determined in a similar manner:

$$C_{FITC} = (A_{495} / (\varepsilon' \times l)) \times DF$$

$$= (0.275\ AU / (73{,}000\ M^{-1}.cm^{-1} \times 1\ cm)) \times 1{,}000$$

$$= 0.0038\ M$$

The molar ratio of FITC label to Nb2 in the Nb2-F sample is therefore 0.0038 M:0.001 M or 3.8:1. Thus, **on average, each mole of Nb2 in the Nb2-F stock has been labelled by 3.8 moles of FITC.**

Calculation S1. Steps used in calculating the degree of Nb2 labelling by FITC. Note that absorbance is measured in arbitrary absorbance units (AU) which are not included in the final calculated concentrations.

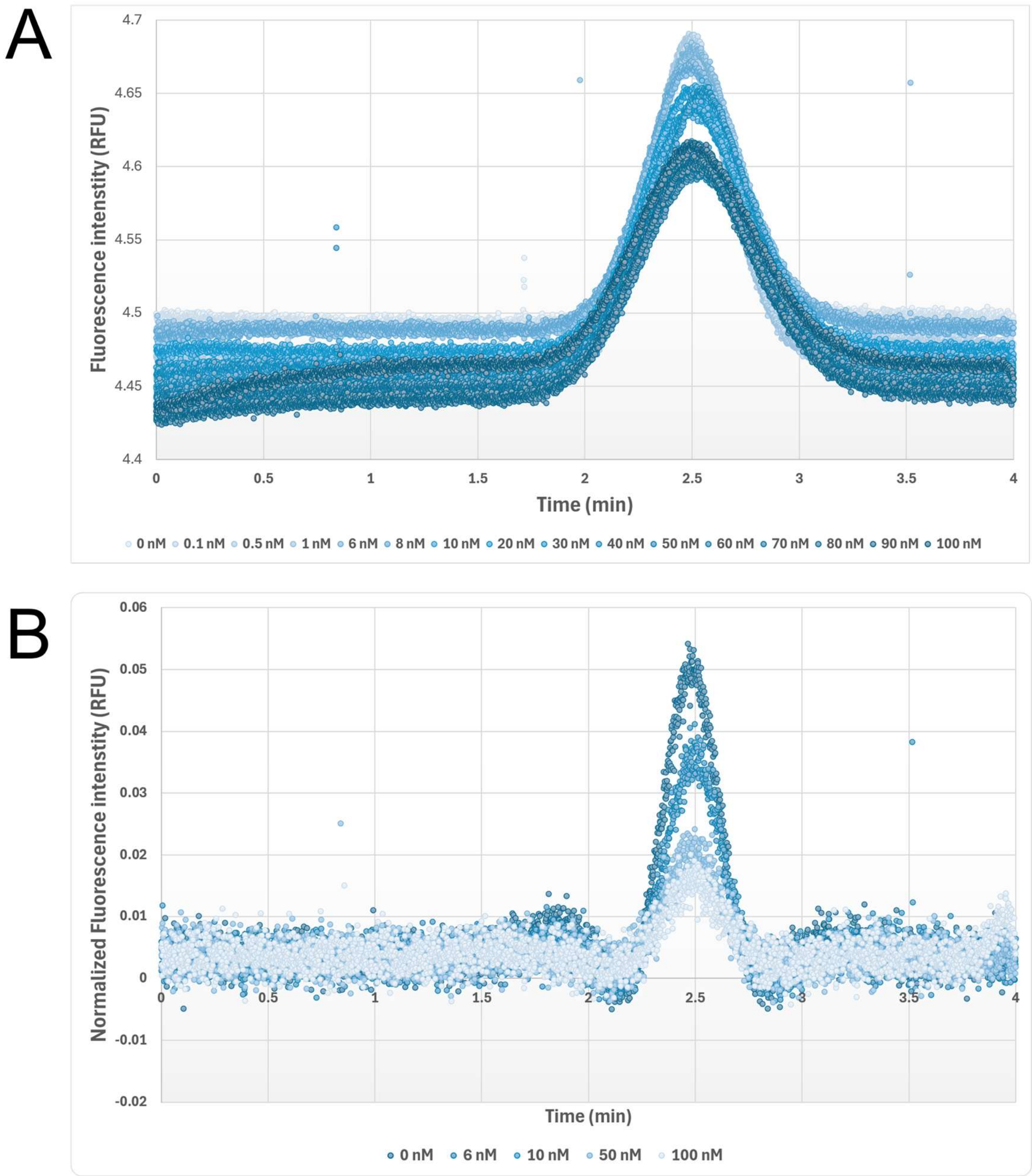

Fig. S4. (A) Representative raw dispersion profiles for samples containing 50 nM Nb2-F equilibrated with the ABC transporter protein at concentrations from 0 nM to 100 nM. Measurements were recorded at 10 °C. (B) Representative dispersion profiles after normalization of baselines. A select set of normalized profiles are shown to minimise overlap and aid visualization.

Table S1. Hydrodynamic radius (Rh) calculated for particles in samples containing 50 nM Nb2-F and 0 to 100 nM ABC transporter protein.

ABC (nM)	Rh (nm)	Std. Dev. (nm)
0	3.18	0.06
0.01	3.16	0.09
0.1	3.23	0.06
0.5	3.22	0.05
1	3.40	0.02
4	3.92	0.05
6	4.27	0.12
8	4.60	0.02
10	4.75	0.04
20	5.29	0.16
30	5.69	0.07
40	5.96	0.35
50	6.32	0.04
60	6.46	0.14
70	6.68	0.16
80	6.76	0.02
90	6.83	0.05
100	6.86	0.29

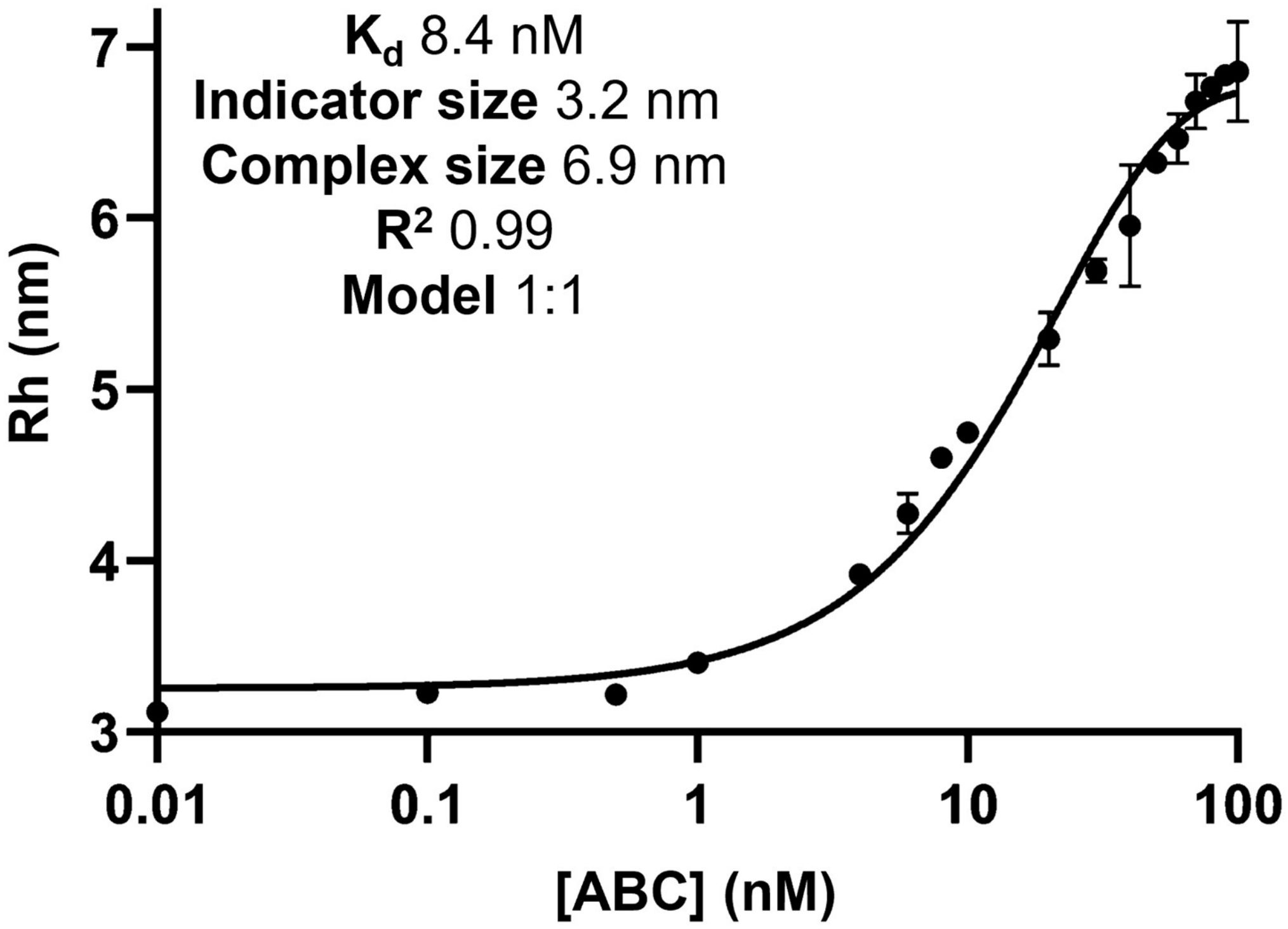

Fig. S5. Binding isotherm derived from FIDA runs at 50 nM Nb-F (indicator) and different ABC transporter (analyte) concentrations with fitting to a 1:1 binding model. Experiments were run in triplicate, and standard deviations are indicated by error bars. Indicator and complex size refer to the Rh measured at the beginning (0 nM ABC) and end (100 nM ABC) of the titration.

References

1. Zimmermann, P. Egloff, C. A. Hutter, B. T. Kuhn, P. Bräuer, S. Newstead, R. J. Dawson, E. R. Geertsma and M. A. Seeger, *Nat. Protoc.*, 2020, **15**, 1707–1741.
2. E. Gasteiger, C. Hoogland, A. Gattiker, S. Duvaud, M. R. Wilkins, R. D. Appel and A. Bairoch, in *The Proteomics Protocols Handbook*, ed. J. M. Walker, Humana Press, New Jersey, 2005, pp. 571–607.
3. C. A. Hutter, M. H. Timachi, L. M. Hürlimann, I. Zimmermann, P. Egloff, H. Göddeke, S. Kucher, S. Štefanić, M. Karttunen, L. V. Schäfer and E. Bordignon, *Nat. Commun.*, 2019, **10**, 2260.
4. D. E. Otzen, A. K. Buell and H. Jensen, *Curr. Opin. Struct. Biol.*, 2021, **70**, 8–15.
5. V. Folcher, R. Auger, C. Louche, P. Serror and T. Touzé, J. Biol. Chem., 2025, 110929.
6. "Quest Database™ Correction Factor [FITC (Fluorescein-5-isothiocyanate)]." *AAT Bioquest, Inc.*, 3 Aug. 2026, https://www.aatbio.com/resources/correction-factor/5_fitc_fluorescein_5_isothiocyanate.
7. M. E. Pedersen, J. Østergaard and H. Jensen, *ACS Omega*, 2020, **5**, 10519–10524.
